# Role of the CX3CL1/CX3CR1 axis in iron metabolism and immune regulation during acute *Trypanosoma cruzi* infection

**DOI:** 10.3389/fimmu.2025.1585883

**Published:** 2025-08-27

**Authors:** Sirlaine Pio, Tatiana Prata Menezes, Vitória Louise, Guilherme de Paula Costa, Daniel Malta Oliveira, Natiele Carlos, Silvia Paula-Gomes, Luiza Oliveira Perucci, André Talvani

**Affiliations:** ^1^ Laboratory of Immunobiology of Inflammation, Department of Biological Science, Federal University of Ouro Preto, Ouro Preto, Minas Gerais, Brazil; ^2^ Graduate Program in Health and Nutrition, Federal University of Ouro Preto, Ouro Preto, Minas Gerais, Brazil; ^3^ Graduate Program in Health Sciences – Infectiology and Tropical Medicine, Federal University of Minas Gerais, Belo Horizonte, Minas Gerais, Brazil; ^4^ Laboratory of Biochemistry and Molecular Biology, Department of Biological Science, Federal University of Ouro Preto, Ouro Preto, Minas Gerais, Brazil; ^5^ School of Medicine, University of California, San Diego, San Diego, CA, United States

**Keywords:** inflammation, *Trypanosoma cruzi*, cardiac disease, CX3CR1, CX3CL1

## Abstract

**Introduction:**

During *Trypanosoma cruzi* infection, the immune system activates a robust inflammatory response, involving cytokines and chemokines like IFN-γ, TNF, IL-6, IL-1β, CCL2, and CCL5, to control parasite replication. The CX3CL1 chemokine and its receptor, CX3CR1, have been implicated in amplifying inflammation through pathways like NF-κB, MAPKs, STATs, TLRs, and NLRs, contributing to tissue damage. This study evaluated the effects of blocking CX3CR1 with the allosteric antagonist AZD8797 in a murine model of acute *T. cruzi* infection.

**Methods:**

Male C57BL/6 mice were infected with 10^3^ trypomastigote forms of *T. cruzi* (Y strain) and received AZD8797 (10 mg/kg) intraperitoneally for 10 days. On the 10th day, animals were euthanized and heart, skeletal muscle, and liver tissues were collected for CX3C L1 protein expression, biomarkers (IL-1β, IL-4, IL-6, IL-10, IL-15, IL-17, IFN-γ, TNF, and CCL2) quantified by Cytometric Bead Array and Enzyme Immunoassay.

**Results:**

Treatment reduced spleen mass and cardiac levels of CCL2 and IL-15, with an increase of IL-4. Conversely, in skeletal muscle, TNF, IL-6, and IL-10 increased, while IL-15 decreased. Liver tissue showed reduced IL-15, IL-6, and IL-1β levels, alongside lowered plasma hepcidin and ferritin concentrations.

**Discussion:**

These findings highlight CX3CL1’s site-specific role in modulating inflammation and iron metabolism during acute *T. cruzi* infection, suggesting its potential as a therapeutic target for infection management and disease prognosis.

## Graphical Abstract

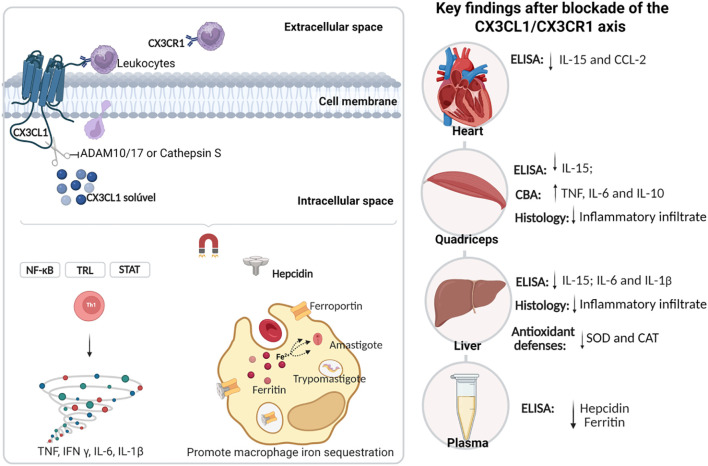

## Introduction

The protozoan *Trypanosoma cruzi* triggers a systemic and multifaceted inflammatory response upon infecting vertebrate hosts, often culminating in chronic cardiomyopathy (1–3). This inflammatory cascade is initiated by parasite molecules, such as glycosylphosphatidylinositol (GPI)-anchored glycoconjugates and extracellular vesicles, which activate macrophages and other immune cells (4, 5). The resulting release of inflammatory mediators across tissues aims to control the parasite but also contributes to disease pathogenesis in both experimental models and humans (6–9).

Among these mediators, CX3CL1 has emerged as a dual-function chemokine, regulating inflammation and enhancing tissue repair (10) while exacerbating *T. cruzi*-induced cardiomyopathy (11). The CX3CL1-CX3CR1 axis has demonstrated therapeutic potential in modulating inflammatory responses in models of atherosclerosis and cardiac hypertrophy, by limiting monocyte recruitment and reducing tissue damage (12, 13). Additionally, this axis plays a role in iron regulation during inflammation, promoting hepcidin production (14, 15). This suggests a possible link between the CX3CL1-CX3CR1 axis, inflammation, and iron metabolism, particularly in cardiac diseases.

In *T. cruzi* infections, intracellular iron sequestration enhances parasite pathogenicity (16–18). Hepcidin, a hepatic hormone that regulates systemic iron levels, is upregulated by inflammatory mediators such as IL-6 and IL-1β (19, 20). Elevated hepcidin levels promote macrophage iron sequestration by degrading ferroportin and increasing ferritin storage (21). *In vitro* studies have shown that CX3CL1 can induce hepcidin expression in microglial cells, suggesting a potential link between this chemokine and iron homeostasis (14).

Despite these insights, the role of the CX3CL1-CX3CR1 axis in immune responses and iron metabolism during *in vivo T. cruzi* infection remains unexplored. This study investigates the modulatory effects of CX3CL1 via allosteric blockade of the CX3CR1 receptor (using AZD8797) on immune responses and iron metabolism during acute infection with the *T. cruzi* - Y strain.

## Materials and methods

### Animals

Twenty-four 10-week-old male C57BL/6 mice, weighing approximately 24 g, were obtained from the Center for Animal Science (CCA) at the Federal University of Ouro Preto (UFOP), Minas Gerais, Brazil. The mice were housed in an air-conditioned room maintained at 22.0 ± 2°C with a 12-hour light/dark cycle and had unrestricted access to water and standard chow. The study protocol was approved by the Ethics Commission on the Use of Animals (CEUA) at UFOP under protocol number 2025080822, in compliance with Resolution 196/96 of the National Health Council of the Brazilian Ministry of Health.

### *Trypanosoma cruzi* infection

Experimental infection was initiated using bloodstream trypomastigote forms of the *T. cruzi* Y strain, harvested from Swiss mice seven days post-infection. The parasites were counted and adjusted in saline solution to a concentration of 10³ parasites per mouse in a 100 µL volume for intraperitoneal injection. Infection was confirmed by parasitemia analysis as described by Brener (22).

### Treatment

**Figure 1:**
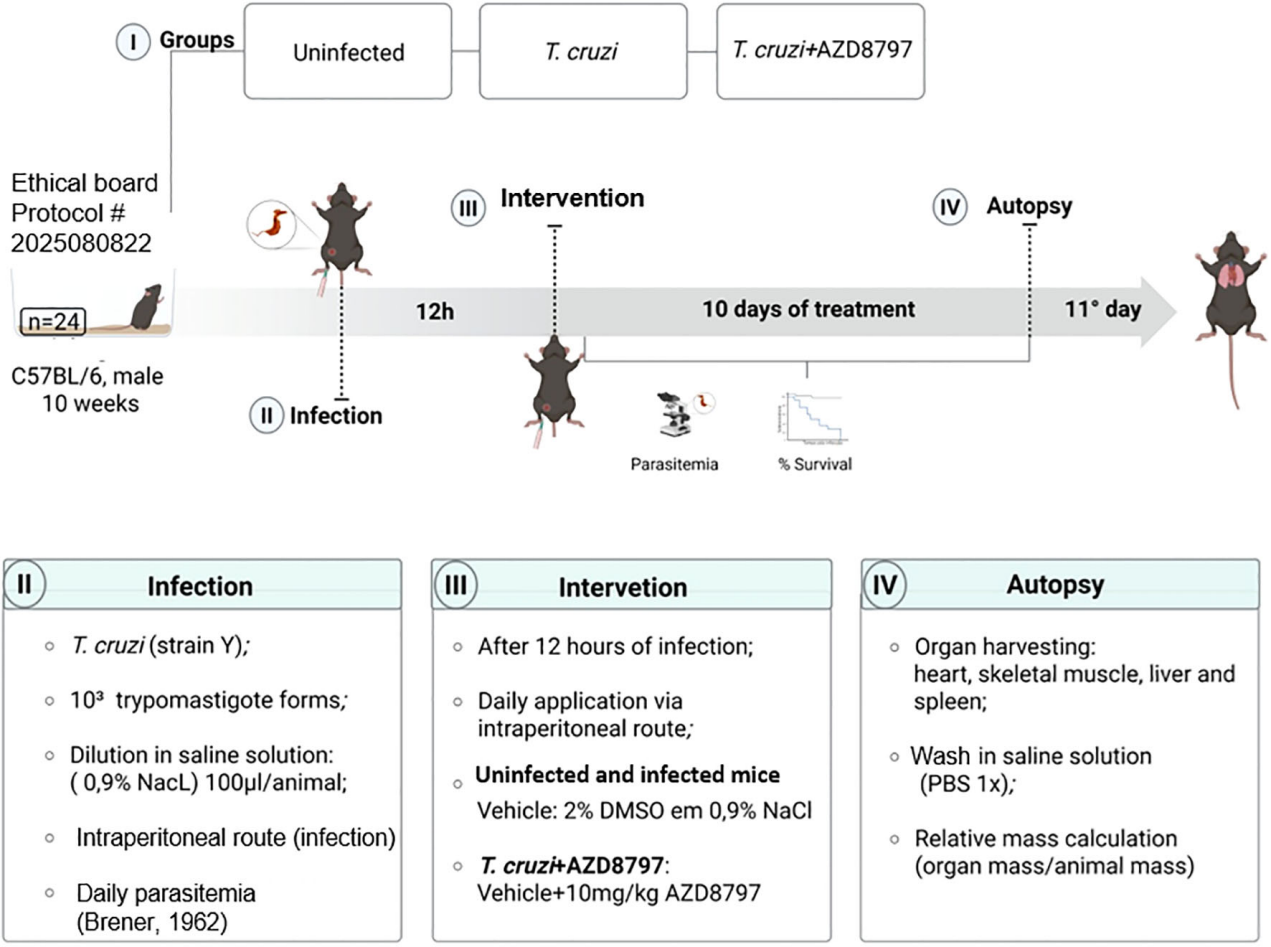
Timeline and experimental design. Male C57BL/6 mice (10 weeks old) were divided into three groups: Uninfected, *T. cruzi*-infected, and *T. cruzi* + AZD8797. Infection was performed using 10³ trypomastigotes (Y strain) via intraperitoneal injection. After 12 hours, treatment with AZD8797 (10 mg/kg/day) or vehicle was initiated and continued for 10 days. Parasitemia and survival were monitored during treatment. On day 11 post-infection, animals were euthanized, and organs (heart, skeletal muscle, liver, and spleen) were collected for analysis.

Infected mice were treated daily with the CX3CL1 receptor antagonist AZD8797 (MedChemExpress, NJ, USA) or its vehicle via intraperitoneal injection. AZD8797 was administered at a dose of 10 mg/kg/day, solubilized in a vehicle solution containing 2% dimethyl sulfoxide (DMSO) in 0.9% NaCl, as per the manufacturer’s instructions. Treatment commenced 12 hours post-infection and continued for 10 consecutive days (**Figure 1**). A parallel uninfected control group received the same volume of either AZD8797 or the vehicle.

### Western blot

Protein expression in heart tissue homogenates was analyzed using standard immunoblotting techniques. Protein concentrations were determined using the method described by Lowry et al. (23). Supernatants were mixed with Laemmli sample buffer (1:1), subjected to electrophoresis on a 10% SDS-PAGE gel, and transferred onto nitrocellulose membranes. The membranes were probed with anti-CX3CL1 (1:1000, #14-7986-81, eBioscience, USA) and anti-β-actin (1:1000, Cell Signaling Technology, MA, USA) primary antibodies. Secondary antibodies conjugated to peroxidase (1:5000, Cell Signaling Technology, MA, USA) were used for detection. Band intensities were quantified using ImageJ2 software (version 1.46m, NIH, USA).

### Cytometric bead array immunoassay

Levels of inflammatory and regulatory cytokines, including IFN-γ, TNF, IL-4, IL-6, IL-10, and IL-17, were quantified using a mouse Th1/Th2/Th17 CBA kit (BD Biosciences, CA, USA). Cytokine levels were measured in supernatants of cardiac and skeletal muscle tissues using a BD FACSCalibur flow cytometer. Data were analyzed with CBA software and expressed in pg/mL, following the manufacturer’s protocol.

### Enzyme-linked immunosorbent assays

Cytokines (IL-6, IL-1β, IL-10, IL-15) and the chemokine CCL2 were quantified in heart, skeletal muscle, and liver homogenates using commercially available ELISA kits (PeproTech^®^, Rocky Hill, NJ, USA). Hepcidin and ferritin levels in plasma samples from infected and treated animals were also measured using ELABScience kits (TX, USA). All procedures were performed in duplicate, adhering to the manufacturers’ instructions. Results were expressed as pg/mL.

### Histological processing and number of infiltrated cells

Fragments of cardiac, skeletal muscle, and liver tissues were collected to assess cellular infiltration. These tissue samples were fixed in 10% buffered formalin for 24 hours, followed by dehydration and embedding in paraffin blocks. Thin sections (5 μm) were obtained using a rotary microtome equipped with a steel blade and subsequently stained with hematoxylin and eosin (HE). The number of infiltrated cells was quantified by analyzing randomly selected fields, covering a total area of 25,000 μm², at 40× magnification. Images were captured using an Axioscope A1 microscope (Carl Zeiss, Germany) and analyzed with ImageJ software (version 6.0, National Institutes of Health, USA).

### Analysis of antioxidant defenses

Cardiac, skeletal muscle, and liver tissues were analyzed for oxidative damage by measuring the activity of key antioxidant enzymes. Superoxide dismutase (SOD) activity was assessed spectrophotometrically at 570 nm, following the method described by Marklund and Marklund (24). As SOD inhibits pyrogallol autoxidation, increased enzymatic activity indicates a reduction in pyrogallol oxidation. Catalase (CAT) activity was measured according to Aebi (25) by monitoring the decrease in hydrogen peroxide (H_2_O_2_) concentration at 240 nm over 60 seconds. Enzymatic activities were expressed as units per milligram of protein (U/mg).

### Statistical analysis

Data analysis was conducted using GraphPad Prism software (v8.3, GraphPad Software, San Diego, CA, USA). Results are presented as mean ± standard deviation (SD). Normality was assessed using the Shapiro-Wilk test. For comparisons between two groups, a one-way Student’s *t*-test was used, and Tukey’s *post hoc* test was applied for multiple comparisons. Statistical significance was set at *p*< 0.05.

## Results

**Figure 2:**
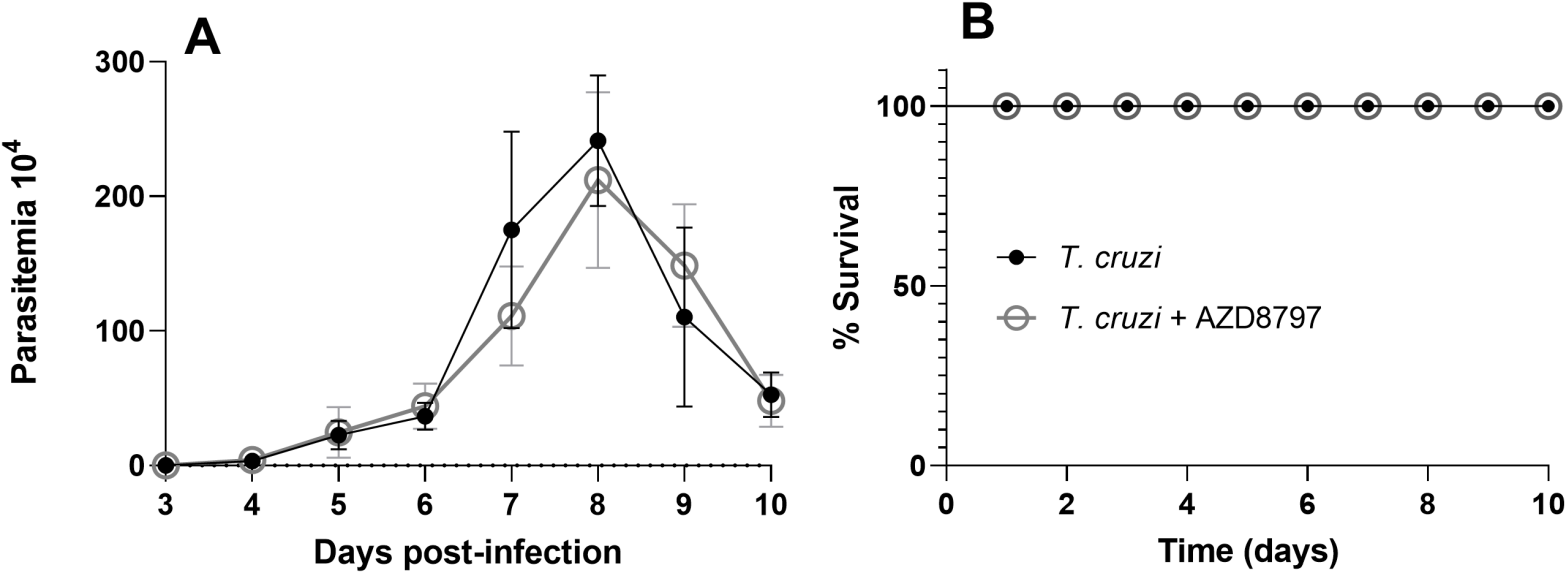
Parasitemia and survival curve. The level of parasitemia under CX3CL1 receptor blockade **(A)**; the survival curve under CX3CL1 receptor blockade in mice infected with the *T. cruzi* Y strain during acute infection **(B)** (n=8). Data expressed as mean ± standard deviation (SD).

In this study, parasitemia was monitored throughout the experiment, reaching its peak on the 8th day post-infection (**Figure 2A**). Daily administration of 10 mg/kg AZD8797 had no significant effect on the survival or mortality curves of infected mice (**Figure 2B**). Furthermore, AZD8797 treatment did not alter the relative mass of the heart or quadriceps muscle. However, *T. cruzi* infection led to a significant increase in the relative spleen mass, from 2.65 g ± 0.17 in uninfected mice to 22.40 g ± 1.54 in infected animals (*p<0.0001*). Notably, AZD8797 administration partially attenuated this increase, reducing the spleen mass of infected mice to 19.12 g ± 1.33 (*p=0.005*).

**Figure 3:**
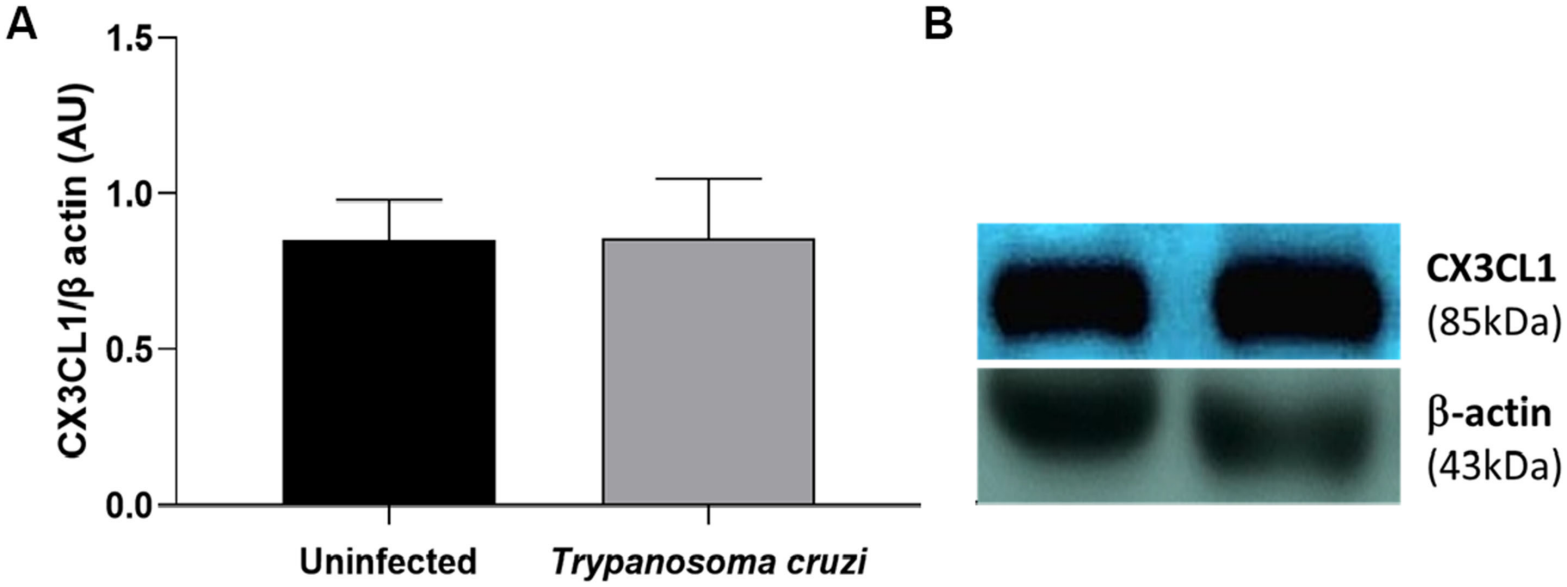
Expression of soluble CX3CL1 in the cardiac tissue. The western blot quantitative analysis of the soluble CX3CL1 protein levels in the cardiac tissue **(A)**; the representative examples of the western blot analysis of the soluble CX3CL1 protein levels in the cardiac tissue **(B)**. The soluble CXC3CL1 were determined by normalization with β-actin. The data are demonstrated as the mean ± standard deviation (SD) (n=6). AU, Arbitrary units.

To assess the cardiac production of the polypeptide CX3CL1 in both uninfected and *T. cruzi*-infected mice, western blot analysis was performed. CX3CL1 expression was found to be preserved and highly expressed in the cardiac tissue of both treated and untreated mice 10 days post-infection (**Figure 3**). A representative gel image depicting band intensities is shown on the right side of the graphic.

**Figure 4:**
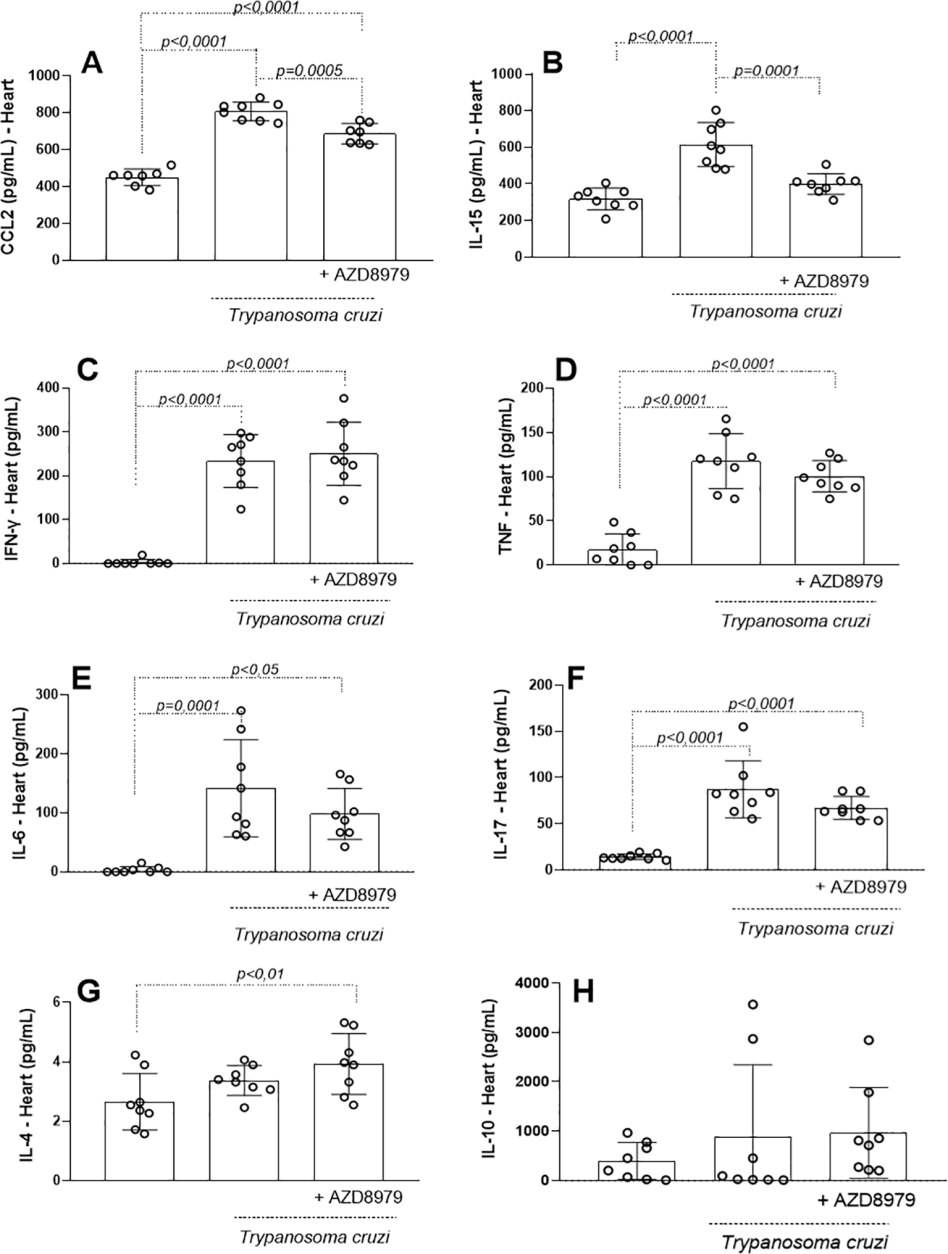
Inflammatory markers in the cardiac tissue. The concentration of inflammatory mediators in the cardiac tissue was detected in uninfected and infected-T*. cruzi* mice treated or not with CX3CL1 receptor blockade during 10 days. Cardiac tissue macerate was evaluated by ELISA as **(A)** CCL2, **(B)** IL-15, and by cytometric bead array (CBA) as **(C)** IFN-γ, **(D)** TNF, **(E)** IL-6, **(F)** IL-17, **(G)** IL-4 and **(H)** IL-10. Data expressed as mean ± standard deviation (SD) (n=8). The *p-values* were determined by one-way ANOVA with Tukey’s posttest and expressed in pg/mL.

After confirming that soluble CX3CL1 was preserved in both uninfected and infected mice, we evaluated the influence of the CX3CL1/CX3CR1 axis on the release of soluble biomarkers in cardiac and skeletal muscle. In the cardiac tissue, CCL2 (**Figure 4A**) and IL-15 (**Figure 4B**) productions were significantly elevated in response to *T. cruzi* infection, but both were reduced following AZD8797 administration. IFN-γ (**Figure 4C**), TNF (**Figure 4D**), IL-6 (**Figure 4E**), and IL-17A (**Figure 4F**) levels were also increased in infected cardiac tissue. However, AZD8797 did not reduce their production. Interestingly, AZD8797 treatment elevated IL-4 levels (**Figure 4G**) compared to uninfected mice, while IL-10 levels (**Figure 4H**) remained unchanged across all groups.

**Figure 5:**
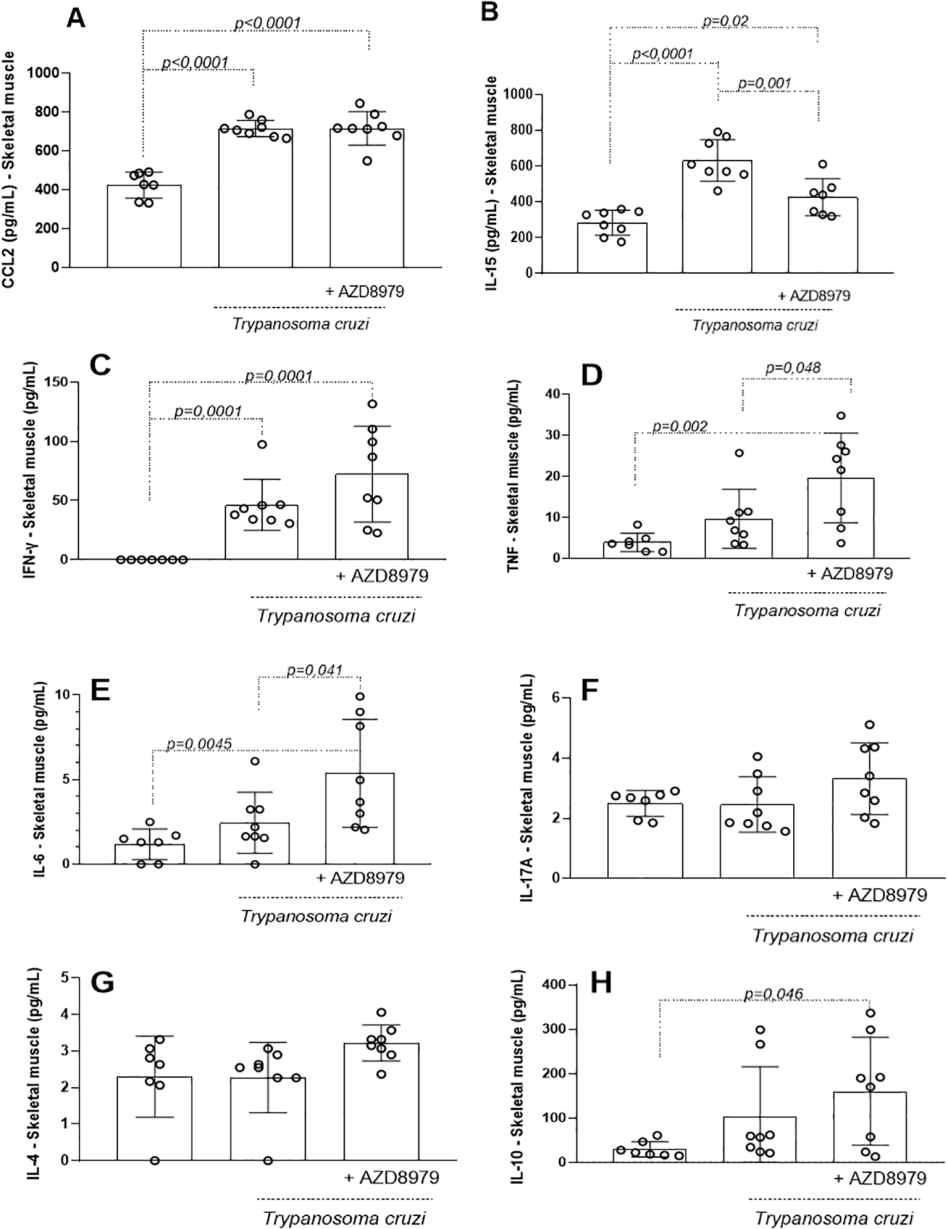
Inflammatory markers in the skeletal muscle. The concentration of the inflammatory markers in the skeletal tissue was detected in uninfected and infected-T*. cruzi* mice treated with CX3CL1 receptor blockade or vehicle for 10 days. Immunoassay was performed as **(A)** CCL2, **(B)** IL-15 and cytometric bead array (CBA) as **(C)** IFN- γ, **(D)** TNF, **(E)** IL-6, **(F)** IL-17, **(G)** IL-4 and **(H)** IL-10. Data expressed as mean ± standard deviation (SD) (n=8). The *p* values were determined by one-way ANOVA with Tukey’s posttest and expressed in pg/mL.

In skeletal muscle, *T. cruzi* infection increased levels of CCL2 (**Figure 5A**), IL-15 (**Figure 5B**), and IFN-γ (**Figure 5C**). AZD8797 administration specifically reduced IL-15 levels. However, during the acute phase of infection, CX3CL1 receptor blockade increased TNF (**Figure 5D**) and IL-6 (**Figure 5E**) production in skeletal muscle. Levels of IL-17A (**Figure 5F**) and IL-4 (**Figure 5G**) were unaffected by infection or CX3CL1 receptor inhibition. Interestingly, IL-10 levels (**Figure 5H**) were significantly elevated in infected mice following AZD8797 treatment.

**Figure 6:**
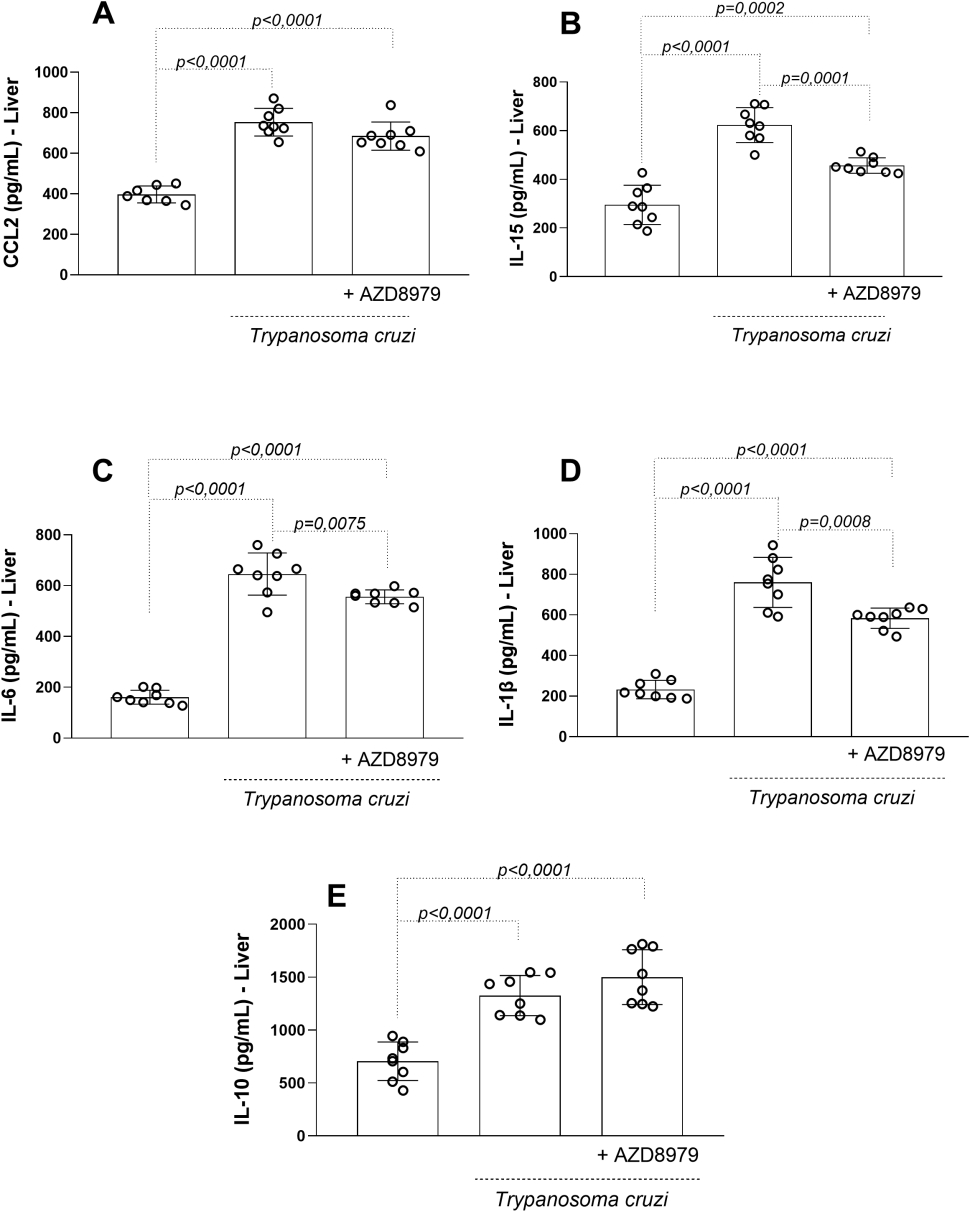
The liver levels of the inflammatory markers. The inflammatory markers **(A)** CCL2, **(B)** IL-15 **(C)** IL-6, **(D)** IL-1β, e **(E)** IL-10 in uninfected and infected *T. cruzi*, under CX3CL1 receptor blockade (or vehicle/control), were measured by enzyme-linked immunosorbent assay. Data expressed as mean ± standard deviation (SD) (n=8). The *p* values were determined by one-way ANOVA with Tukey’s posttest and expressed in pg/mL.

In the liver, *T. cruzi* infection resulted in elevated levels of CCL2 (**Figure 6A**), IL-15 (**Figure 6B**), IL-6 (**Figure 6C**), IL-1β (**Figure 6D**), and IL-10 (**Figure 6E**). AZD8797 treatment effectively reduced the production of IL-15, IL-6, and IL-1β, but not CCL2 or IL-10.

Together with inflammatory markers, the activity of enzymes SOD and CAT were also determined in the cardiac, skeletal muscle and liver to verify alterations in the antioxidant defense system. The SOD and CAT (**Table 1**) was higher in animals infected. The blockade of the CX3CL1 receptor via AZD8797 administration reduced both enzymes in the liver. However, there is an increased CAT in the skeletal muscle when using the blockade of the CX3CL1 receptor.

**Table 1:** Oxidative stress markers in cardiac and skeletal muscles and, in liver.

	Tissues	Uninfected	*T.cruzi*	*T.cruzi*+AZD8979	p^1^	p^2^	p^3^
**SOD** **(U/mg de protein)**	**Cardiac**	5.383±0.53	6.298±0.44	6.021±0.60	0.0068**	0.0652	0.5614
	**Skeletal**	4.760±0.48	6.091±0,55	5.625±0.52	0.0001***	0.0091**	0.2003
	**Hepatic**	2.399±0.28	3.400±0.33	2.930±0.38	<0.0001****	0.0128*	0.0286*
**CAT** **(U/mg de protein)**	**Cardiac**	6.030±1.81	9.827±1.26	7.865±1.56	0.0002***	0.0705	0.0510
	**Skeletal**	1.800±0.78	3.010±0.77	3.271±1.51	0.0888	0.0334*	0.8808
	**Hepatic**	8.415±1.31	13.38±3.29	8.925±0.96	0.0004***	0.8811	0.0011**

SOD, superoxide dismutase; CAT, catalase. Data expressed as mean ± standard deviation (SD) (n=8). The P values were determined by one-way ANOVA with Tukey's posttest and expressed in U/mg de protein *p≤0,05. p^1^: Uninfected x *T. cruzi;* p^2^: Uninfected x T. cruzi+AZD8797; p^3^: *T. cruzi* x *T. cruzi*+AZD8797. *p< 0.05, **p< 0.01, ***p< 0.001 and ****p< 0.0001.

**Figure 7:**
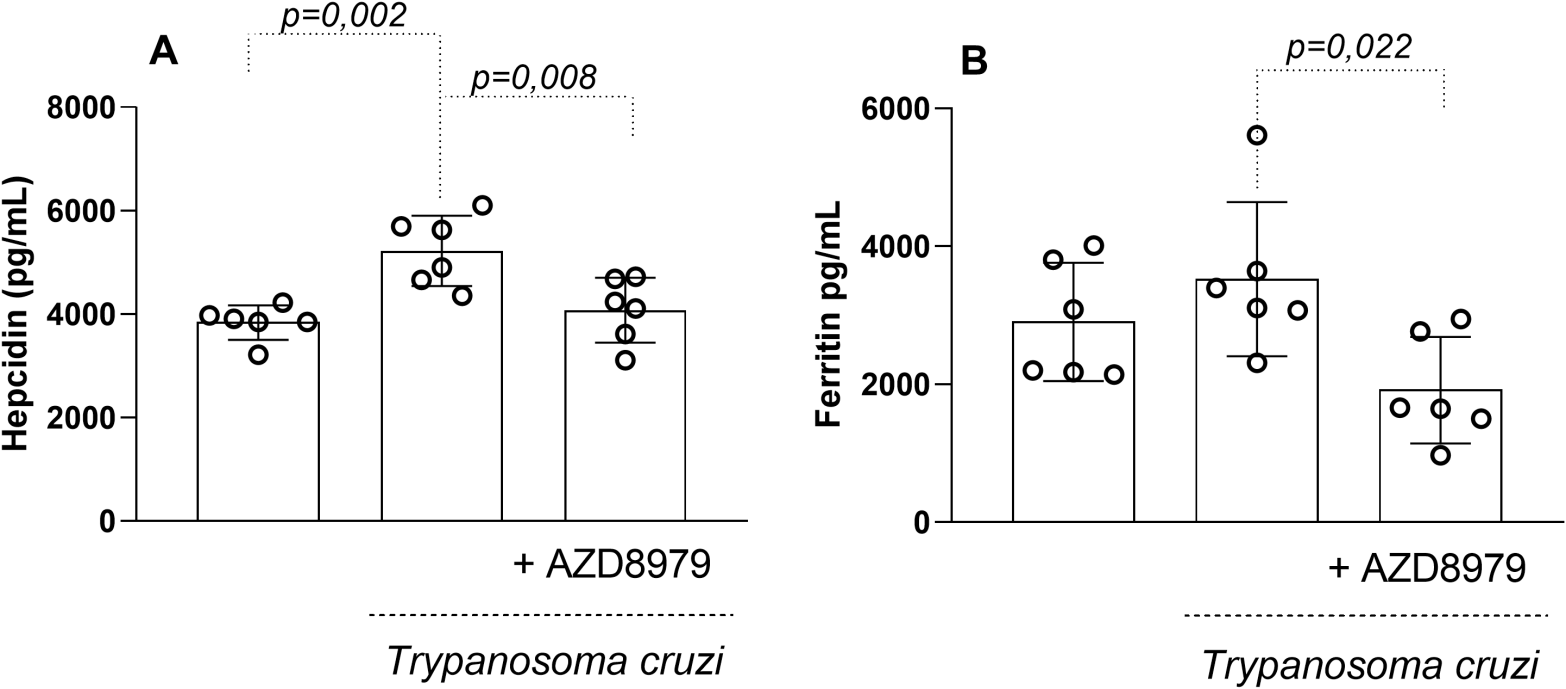
The serum levels of hepcidin and ferritin. The serum levels of the hepcdina **(A)**, ferritina **(B)** in uninfected and *T. cruzi*-infected mice treated with CX3CL1 receptor blockade were measured by enzyme-linked immunosorbent assay. Data expressed as mean ± standard deviation (SD) (n=8). The *p-values* were determined by one-way ANOVA with Tukey’s posttest and expressed in pg/mL.

In this study, the CX3CL1/CX3CR1 axis was also evaluated contributing to ferric modulation. Infected mice exhibited significantly increased serum levels of hepcidin (**Figure 7A**) and ferritin (**Figure 7B**) during the acute phase of infection. Blockade of the CX3CL1 receptor via AZD8797 administration effectively reduced serum ferric element levels, highlighting a modulatory effect on iron metabolism during *T. cruzi* infection.

**Figure 8:**
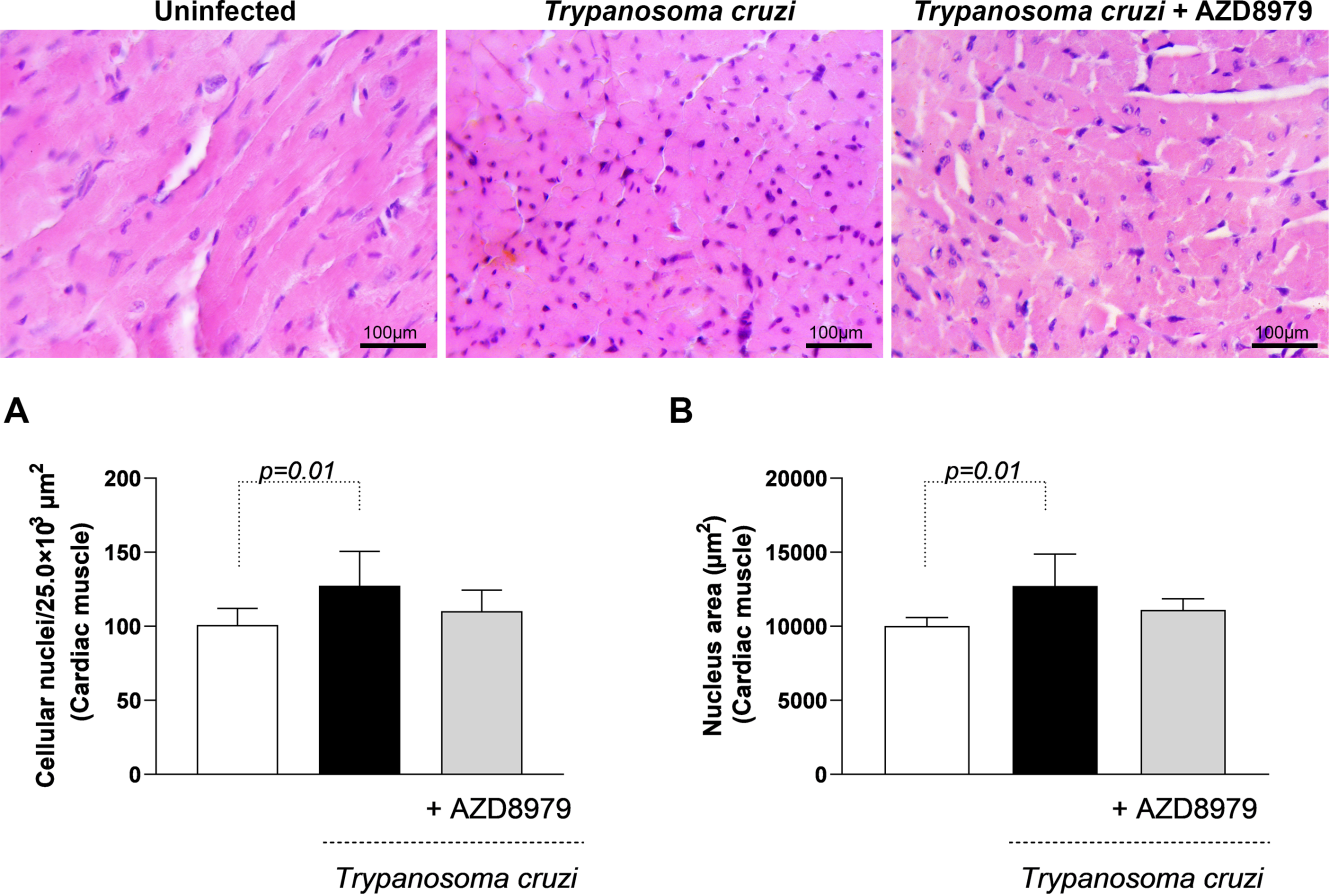
Morphological analysis of inflammatory infiltrate in cardiac muscle tissue. **(A)** Quantification of the number of cell nuclei per 25.0x10³ µm² of cardiac tissue; **(B)** Mean nuclear area (µm²) in cardiac tissue. Images of cardiac muscle tissue stained with H&E. Bar = 100µm, 400x magnification. Data were expressed as mean ± standard deviation and the p values ​​(p<0.05) were determined by one-way ANOVA with Tukey’s posttest.

**Figure 9:**
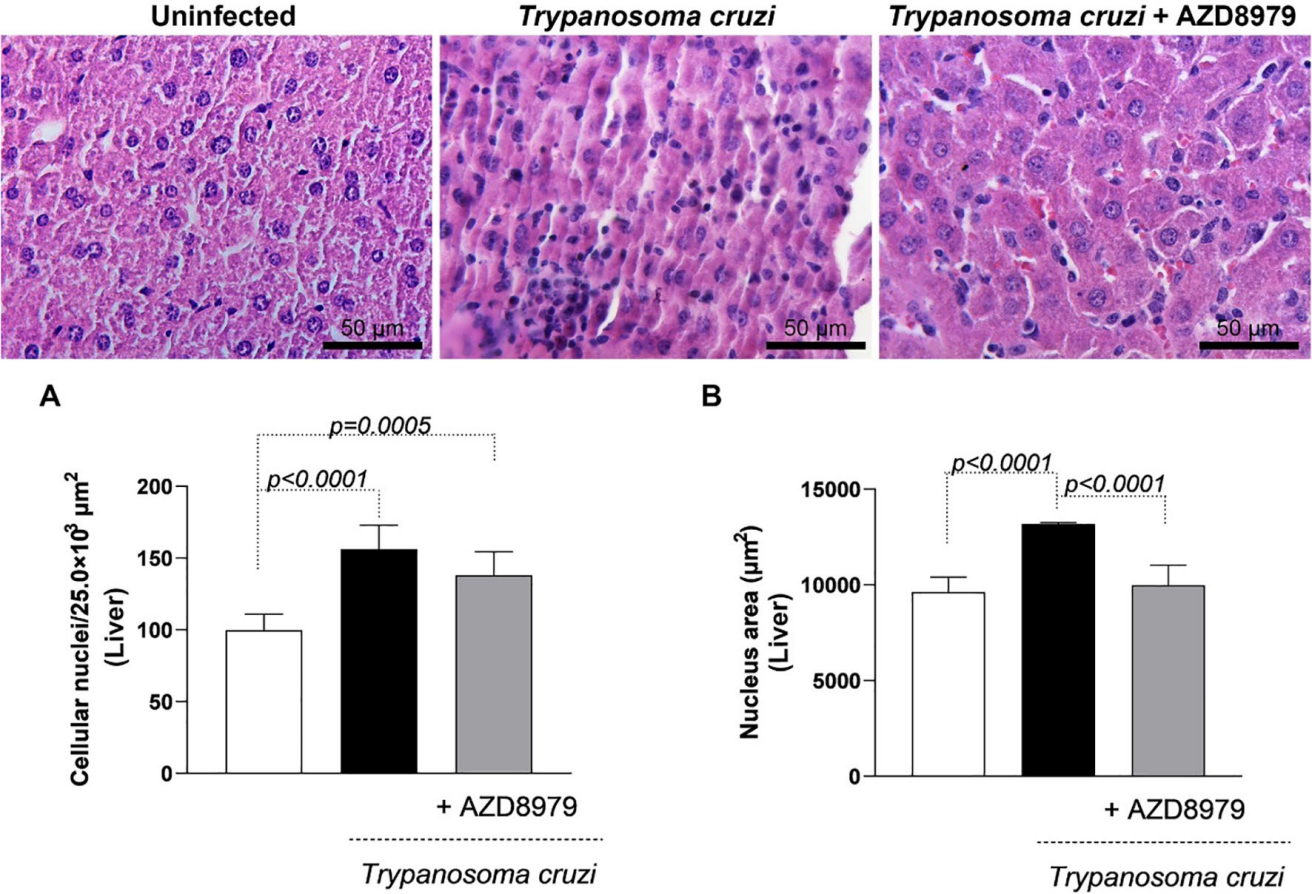
Morphological analysis of the inflammatory infiltrate in the liver. **(A)** Quantification of the number of cell nuclei per 25.0x10³ µm² of liver tissue; **(B)** Mean nuclear area (µm²) in liver tissue. Images of liver tissue stained with H&E. Bar = 50µm, 400x magnification. Data were expressed as mean ± standard deviation and the *p* values ​​(*p*<0.05) were determined by one-way ANOVA with Tukey’s posttest.

**Figure 10:**
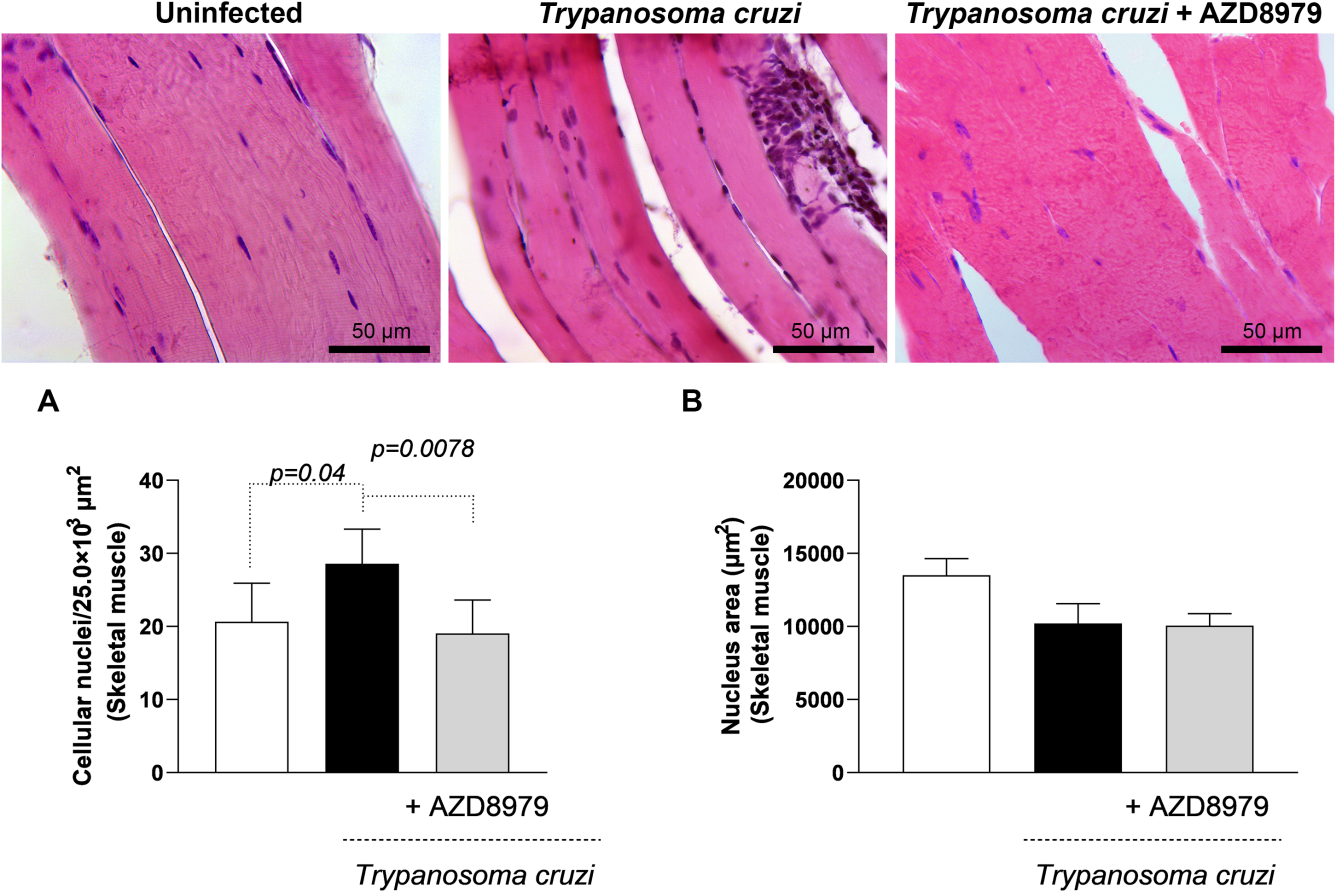
Morphological analysis of inflammatory infiltrate in skeletal muscle. **(A)** Quantification of the number of cell nuclei per 25.0x10³ µm² of skeletal muscle tissue; **(B)** Mean nuclear area (µm²) in skeletal muscle tissue. Images of skeletal muscle tissue stained with H&E. Bar = 50µm, 400x magnification. Data were expressed as mean ± standard deviation, and the *p* values <0.05 were determined by one-way ANOVA with Tukey’s posttest.

It is important to note that this study was conducted during the acute phase of experimental *T. cruzi* infection. Despite this, cardiac, skeletal muscle, and liver tissues were analyzed to quantify inflammatory infiltration following AZD8979 administration. *T. cruzi* infection led to an increase in the number of cellular nuclei (**Figure 8A**) and nucleus area (**Figure 8B**) in cardiac tissue. Interestingly, in liver tissue, *T. cruzi* infection resulted in both an increased number of cellular nuclei (**Figure 9A**) and an expanded nucleus area (**Figure 9B**). Treatment with the CX3CL1R blocker successfully reduced both inflammatory parameters. Finally, in skeletal muscle, infection also increased the number of cellular nuclei; however, AZD8979 effectively regulated this inflammatory response (**Figure 10A**), though it did not alter nucleus area (**Figure 10B**).

## Discussion

The observed tissue-specific effects of CX3CR1 inhibition likely reflect the distinct immunological microenvironments and differential parasite loads in different tissues during acute *T. cruzi* infection, even without altering blood parasitemia. The heart, a major target of *T. cruzi*, typically sustains high levels of parasite persistence and inflammation, which may explain why CX3CR1 blockade led to a significant reduction in proinflammatory mediators such as CCL2 and IL-15, along with an increase in the anti-inflammatory cytokine IL-4. This suggests a potential protective modulation of cardiac inflammation. In contrast, the skeletal muscle exhibited elevated levels of TNF, IL-6, and IL-10 following treatment, indicating a shift toward a more complex inflammatory profile, suggestive of a compensatory response in this less severely affected tissue. Meanwhile, the liver, the main site of iron homeostasis and of *T. cruzi*-induced inflammation, showed a marked reduction in IL-6, IL-1β, and IL-15, consistent with the observed decline in circulating hepcidin and ferritin. These changes suggest that CX3CR1 signaling may contribute to hepatic inflammation and iron regulation during infection. These findings underscore a multifaceted and compartmentalized nature of C57BL/6 mice in response to *T. cruzi*, and highlight how CX3CL1/CX3CR1 axis modulation can yield divergent outcomes depending on tissue-specific dynamics of immune activation, parasite burden, and homeostatic demands.

Iron (Fe) is a critical micronutrient for *T. cruzi* and other trypanosomatids like *Leishmania* spp. However, the role of Fe in the life cycle and pathogenicity of these parasites remains incompletely understood. *T. cruzi* depends on Fe for vital metabolic functions, including DNA synthesis, heme biosynthesis, energy production via the electron transport chain, oxidative phosphorylation, and pathogenicity in mammalian hosts (16). The parasite hijacks host Fe-proteins to fulfill its metabolic needs. Within the host, free Fe must be stored or processed upon entering the cytosol to prevent the generation of reactive oxygen species, which can cause cellular damage (26). Previous *in vivo* evidence has suggested a key role for iron in providing energy for the *T. cruzi* life cycle and in the propagation of Chagas disease (27). In addition, Estevam and colleagues demonstrated, using portable X-ray fluorescence, that C57BL/6 mice exhibit reduced skin iron levels during acute infection, which correlates with lower parasite burden and increased oxidative stress (28). Notably, the parasite utilizes specific iron transporters, such as the 39-kDa protein TciT and the iron reductase TcFR, to internalize iron (29). There is growing evidence that TciT and TcFR may also participate in copper transport and intracellular distribution, another essential element for aerobic metabolism in trypanosomatids (30). These findings support the proposed role of iron regulation in determining disease outcomes and should be considered to strengthen the mechanistic rationale of iron involvement in *T. cruzi* infection.

During *T. cruzi* infection, CX3CL1 appears to extend its role beyond leukocyte recruitment, promoting increases in ferritin and hepcidin levels. Hepcidin, a key iron-regulatory hormone, modulates the body’s iron balance and plays a significant role in innate immunity by regulating Fe availability during infections (31). Ferritin, synthesized by nearly all living organisms, serves as a key intracellular and extracellular Fe storage protein, releasing Fe in a controlled manner to prevent toxicity (32). Disruption of the hepcidin/ferritin axis can increase host susceptibility to *T. cruzi* infection.

Under normal physiological conditions, the liver serves as the primary iron reservoir. However, iron is also distributed throughout other organs, where it is integrated into enzymes and structural proteins, particularly in the skeletal muscles, pancreas, brain, and heart. Given the close relationship between iron and hemoglobin in red blood cells, and considering that *T. cruzi*-induced inflammation enhances local tissue activity and blood flow, we hypothesize that CX3CR1 blockade may exertxorgan-specific effects on inflammatory cells activation and inflammatory mediator’s release. These effects are likely influenced by both the tissue microenvironment and the genetic background of the infecting *T. cruzi* strain (33, 34).

Previous studies have shown that elevated iron stores correlate with increased parasitemia and higher mortality in mice, a condition reversible by Fe chelation (16). Notably, the efficacy of benznidazole, the clinical drug of choice for treating Chagas disease, is enhanced in the presence of the Fe chelator desferrioxamine (18). Our findings suggest that, during the initial stage of *T. cruzi* infection, blocking the CX3CL1/CX3CR1 axis partially disrupts Fe metabolism, creating an environment less favorable for parasite survival and reducing its pathogenicity.

CX3CL1 is predominantly expressed by endothelial cells in myocardial ischemia and heart failure. It is an atypical chemokine that exists in a membrane-bound form and a cleaved soluble form (35). The membrane-bound form functions as an adhesion molecule, facilitating leukocyte adhesion to NK cells, monocytes, and specific T cell subsets via interaction with its receptor, CX3CR1. Since the CX3CL1/CX3CR1 axis is primarily expressed on endothelial and cardiac cells, it has been implicated in the athogenesis of diseases such as atherosclerosis and heart failure. Notably, the CX3CL1/CX3CR1 axis has been studied in coronary artery disease, where patients exhibit an expansion of the CX3CR1+CD3+CD8+ T cell subset, characterized by enhanced chemotactic, adhesive, and inflammatory responses to CX3CL1 (36).

In the context of cardiomyopathy induced by *T. cruzi* infection, the role of the CX3CL1/CX3CR1 axis was not explored until the late 1990s. Prior to this, other chemokines such as CCL2, CCL5, CXCL9, and CXCL10, along with their receptors, were well-documented as mediators of experimental pathogenesis and contributors to the worsening of clinical status in humans (37–39).

The involvement of CX3CL1 in *T. cruzi* infection was first highlighted in a model of low-protein diet-induced immunomodulation. Intriguingly, systemic CX3CL1 levels were elevated, but cardiac tissue levels were reduced, accompanied by decreased inflammatory infiltration, particularly of CD68+ and CD163+ macrophages (40). At the time, the differential inflammatory responses mediated by CX3CL1 across various tissues did not attract significant attention. However, in a subsequent study using a CX3CR1 inhibitor (AZD8797), distinct inflammatory profiles were observed in the cardiac, hepatic, and skeletal muscle tissues in response to *T. cruzi* infection. These findings are particularly relevant, given that advanced Chagas cardiomyopathy in humans is characterized by dilated cardiomyopathy, reduced ejection fraction, and ventricular enlargement (41).

A subsequent study reinforced the significance of CX3CL1 in *T. cruzi* infection. It demonstrated that intravenous administration of *T. cruzi*-derived neurotrophic factors in MyD88-knockout mice (deficient in TLR signaling) increased CX3CL1 and CCL2 levels, modulating inflammatory responses mediated by Trk signaling and reducing cardiac fibrosis (11). Another study investigating AngII-induced hypertension in the context of *T. cruzi* infection revealed a synergistic effect of hypertension and *T. cruzi* infection on TNF and CX3CL1 expression, leading to enhanced leukocyte recruitment into cardiac tissue (42). Interestingly, TNF and endothelin-1 are well-established biomarkers of pathogenesis in experimental *T. cruzi* infection and indicators of severity in Chagas cardiomyopathy (37, 43–45). The CX3CL1/CX3CR1 axis positively correlates with these biomarkers, exacerbating cardiac pathology in *T. cruzi* infection (11).

In a non-parasitic model, early β-adrenergic stimulation was shown to activate the cardiac CX3CL1/CX3CR1 axis, supporting transient concentric remodeling and delaying the progression to heart failure (46). This finding is relevant to Chagas cardiomyopathy, as adrenergic autoantibodies are known to contribute to the progression of disease (47–49). CX3CL1 and TNF, secreted by inflammatory and cardiac cells, may synergistically promote cardiomyocyte hypertrophy in chronic *T. cruzi* infection.

Altogether, there remains a promising avenue of investigation into the inhibition of the CX3CR1/CX3CL1 axis in the context of Chagas disease. Although clinical application in humans would require a carefully staged development process, this strategy represents a novel and rational shift from traditional antiparasitic multitherapy toward a more integrated immune-metabolic modulation. During *T. cruzi* infection, inflammation-driven upregulation of hepcidin promotes iron sequestration, particularly in macrophages and hepatic tissue, which may create a favorable environment for parasite persistence. The CX3CR1 inhibitor AZD8797 emerges as a potential host-targeted adjunctive therapy by disrupting this iron retention and concurrently mitigating tissue-specific inflammation, notably within the heart. Existing chemotherapeutic options for Chagas disease frequently fail to effectively control parasite replication during both the acute and chronic phases, which negatively impacts long-term cardiac outcomes (50). However, before advancing to human trials with AZD8797, it is critical to conduct comprehensive studies to determine human-specific dose-response relationships and assess the long-term safety of this approach, particularly given the potential effects on iron metabolism and the partial immune suppression associated with CX3CR1 inhibition.

In summary, our findings underscore the CX3CL1/CX3CR1 axis as a critical regulator at the intersection of immune and iron metabolism responses during *T. cruzi* infection. By influencing both inflammatory pathways and iron availability, both key factors in parasite persistence and tissue damage, this axis emerges as a strategic node for therapeutic intervention. Targeting this pathway may not only attenuate pathogenic inflammation but also limit iron-dependent parasite survival, offering a dual mechanism of action in the management of *T. cruzi*-induced heart disease.

## Data Availability

The immunological and biochemical data used to support the findings of this study are available from the corresponding author upon request.
